# Assessment of the Different Type of Materials Used for Removing Phosphorus from Wastewater

**DOI:** 10.3390/ma14164371

**Published:** 2021-08-04

**Authors:** Claudiu Cepan, Adina-Elena Segneanu, Oana Grad, Maria Mihailescu, Melinda Cepan, Ioan Grozescu

**Affiliations:** 1 Department of Applied Chemistry and Engineering of Inorganic Compounds and the Environment, University Politehnica Timisoara, 300006 Timisoara, Romania; cepanclaudiu@gmail.com (C.C.); oanagrad88@yahoo.com (O.G.); mihailescumia@gmail.com (M.M.); cepan.melinda@gmail.com (M.C.); ioangrozescu@gmail.com (I.G.); 2 Faculty of Chemistry, Biology, Geography, West University of Timisoara, 300223 Timisoara, Romania

**Keywords:** pollution, phosphorus, wastewater treatment, nutrient, adsorbents, EDS spectroscopy

## Abstract

Reducing the costs associated with water management, improving water quality and the environment are fundamental requirements of sustainable development. Maintaining the optimal level of phosphorus has a direct impact on water quality and the biological system. Current methods used in tertiary wastewater treatment for phosphorus removal present several disadvantages that influence the final water processing cost. Therefore, it is essential for water quality and food safety to develop ecological, cheap and highly efficient materials. This study reported the first comparative assessment of three different types of materials (magnetic, semiconductors and composite) as environmentally friendly, cheap adsorbents for phosphorus removal from wastewater. Several experiments were done to investigate the influence of adsorbent type, dosage and contact time on the efficiency of the processes. The adsorption process was fast and equilibrium was reached within 150 min. We found that the phosphorus adsorption efficiency on of these materials was higher than the chemical method. The obtained results indicated that specific surface area directly influences the performance of the adsorption process. EDS analysis was used to analyze adsorbents composition and analyze the type and content of elements in the substrate before and after reaction with wastewater.

## 1. Introduction

The constant growth of the world’s population, the expansion of urban areas and excessive industrialization including agriculture are just some of the most important causes that have had a strong negative impact on non-renewable natural resources and the environment, dramatically affecting water quality, soil productivity, security food and quality of life.

According to a recent estimation, about one-fifth of the world’s population has limited water resource. The pollutants in surface waters come mostly from industrial activity: heavy metals, dyes, fats, antibiotics, hormones and other pollutants [1,2,3,4,5,6,7,8,9,10,11,12,13,14,15,16,17,18,19,20,21,22,23,24].

Water management and sustainable management of natural resources directly influences the quality of life and food security. In this context, remedying water problems and streamlining water resources to protect natural ecosystems is a necessity [1,2,25,26,27,28,29,30,31,32,33].

In recent years, mainly due to anthropological activities, there has been a continuous decline in non-renewable natural resources and an increase in environmental damage (water quality, soil productivity), both reflected implicitly in food security. 

However, the prevention of the food crisis by expanding intensive industrialized agriculture and excessive soil fertilization (over 50% more than necessary) have affected the natural ecosystem.

Phosphorus and nitrogen are water pollutants with a negative impact on the biological diversity of ecosystems but have a positive effect on agricultural production [1,2,6,8,9,11,12,15,16,17,18,19,20,21,22,23,24,25,26,27,28,29,30,31,32,33,34,35,36,37,38,39,40,41,42,43,44,45,46,47,48,49,50,51,52,53,54,55,56,57,58,59,60,61,62,63,64,65].

The discharge of these pollutants in excessive amounts causes eutrophication of water, which manifests, at first, in overgrown algae, aquatic plants and cyanobacteria. That water becomes hypoxic and reduces the natural habitat of fish. The process is then followed by the decomposition of flora with the production of carbon dioxide and decreasing pH, which affects the growth and development of the respective fauna (fish, molluscs).

Eutrophication is a phenomenon that affects the ecosystem, as well as economic, social and recreational activities, over time.

The substantial decrease effect of modern agricultural industrialization make it possible to design and develop sustainable technologies for efficient recovery and recycling of the nitrogen and phosphorus from wastewater [1,3,6,8,9,11,12,15,17,18,19,20,21,22,23,24,25,26,27,28,29,30,31,32,33,34,35,36,37,40,41,42,43,44,45,46,47,48,49,50,51,52,53,54,55,56,57,58,59,60,61,62,63,64,65].

In the last half-century, the concentration of phosphorus in wastewater that has reached effluents has reached alarming levels, causing deterioration in water quality. Its presence causes many water quality problems, including increased purification costs, decreased leisure and conservation value of accumulation, animal losses, and the possibly lethal effect of algae toxins in drinking water [4,6,8,11,12,13,14,15,16,17,18,19,20,21,22,23,24,25,26,34,35,36,37,38,44,45,46,47,48,49,50,51,52,53,54,55,63,64,65,66,67,68,69].

It is noteworthy that phosphorus is an essential, non-renewable resource, which cannot be produced or replaced by chemicals and which has a worldwide price that is trending upward due to the expansion of agricultural demand and limited supply.

On the other hand, worldwide, the phosphorus consumption to fertilize soil represents approximately 85% (15 million tons/year) of the entire amount of extracted phosphate rock (Ca_5_(PO_4_)_3_(F,Cl,OH)), and it is appreciated that the globally natural reserves will be depleted in the next 50–100 years [5].

It is estimated that a period of about 10–15 million years to regenerate the natural reserve of phosphorus is necessary [1,2,3,4,6,8,9,10,11,12,13,14,15,16,17,18,19,20,21,22,23,24,25,26,27,28,29,30,31,32,33,34,35,36,37,38,39,40,41,42,43,44,45,46,47,48,49,50,51,52,53,54,55]. The main sources of phosphorus production are of the biotic or abiotic types (fertilizers), which contribute to the increase of its concentration in the water reserves and implicitly to their eutrophication. Controlling the concentration of phosphorus discharged from municipal and industrial wastewater treatment plants is a key factor in preventing eutrophication of surface water.

Municipal wastewater has an average total content of 5–20 mg/L phosphorus, in which 1–5 mg/L has an organic nature and the rest of it has an inorganic nature [1,2,3,4,6,8,9,10,11,12,13,14,15,16,17,18,19,20,21,22,23,24,25,26,27,28,29,30,31,32,33,34,35,36,37,38,39,40,41,42,43,44,45,46,47,48,49,50,51,55,65,66,67,68,69].

Consequently, it is necessary to develop and improve methods of phosphorus removal [1,2,3,4,5,6,7,8,9,10,11,12,13,14,15,16,17,18,19,20,21,22,23,24,25,26,27,28,29,30,31,32,33,34,35,36,37,38,39,40,41,42,43,44,45,46,47,48,49,50,51,55,65,66,67,68,69].

Phosphorus removal can be achieved by physical (membrane separation technologies), chemical or biological methods [1,2,3,4,5,6,7,8,9,10,11,12,13,14,15,16,17,18,19,20,21,22,23,24,25,26,27,28,29,30,31,32,33,34,35,36,37,38,39,40,41,42,43,44,45,46,47,48,49,50,51,55,65,66,67,68,69].

The most commonly used method is the chemical precipitation of phosphorus with iron and aluminum salts, or lime. However, chemical dephosphorization results in high operational costs and increases the volume of sludge by over 40% [1,2,3,4,5,6,7,8,9,10,11,12,13,14,15,16,17,18,19,20,21,22,23,24,25,26,27,28,29,30,31,32,33,34,35,36,37,38,39,40,41,42,43,44,45,46,47,48,49,50,51,55,65,66,68,69].

The disadvantage of chemical precipitation consists of reagent consumption, the cost of which varies. Moreover, the phosphate recovery from the resulting sewage sludge is not effective yet [70,71].

Phosphorus removal can be performed using a biological method or combined with a chemical treatment. Compared to the chemical removal of phosphorus, biological phosphorus removal is more advantageous, efficient and ecological but leads to the formation of large quantities of sludge whose further processing is reflected in the cost of wastewater treatment [1,2,3,4,5,6,7,8,9,10,11,12,13,14,15,16,17,18,19,20,21,22,23,24,25,26,27,28,29,30,31,32,33,34,35,36,37,38,39,40,41,42,43,44,45,46,47,48,49,50,51,55,65,66,67,68,69].

Worldwide, intense efforts are being made to identify high-performance and efficient materials that will ensure not only the reduction of water treatment costs but also the reuse of nutrients recovered from wastewater [1,2,3,4,5,6,7,8,9,10,11,12,13,14,15,16,17,18,19,20,21,22,23,24,25,26,27,28,29,30,31,32,33,34,35,36,37,38,39,40,41,42,43,44,45,46,47,48,49,50,51,55,65,66,67,68,69].

At present, several more or less efficient wastewater treatment technologies are known and applied in the world, which, in most cases, often affect water quality and, moreover, do not allow the removal of nutrients. In this respect, we can mention the following methods: water chlorination, ozonation, different types of membranes, osmosis, etc. [1,2,3,4,5,6,7,8,9,10,11,12,13,14,15,16,17,18,19,20,21,22,23,24,25,26,27,28,29,30,31,32,33,34,35,36,37,38,39,40,41,42,43,44,45,46,47,48,49,50,51,55,65,66,67,68,69].

The latest studies in the field of water treatment have focused on the development of nanotechnology, which are estimated to have a major impact in the very near future [26,27,66]. Due to their special properties, the field of use of nanoparticles is extremely diverse: environmental protection, medicine, IT, energy, biotechnology, agriculture, construction, etc.

The implications of developing efficient nanotechnology for the tertiary stage of wastewater treatment are particularly promising, especially due to the minimization of the number of by-products resulting from the process [1,2,3,4,5,6,7,8,9,10,11,12,13,14,15,16,17,18,19,20,21,22,23,24,25,26,27,28,29,30,31,32,33,34,35,36,37,38,39,40,41,42,43,44,45,46,47,48,49,50,51,55,65,66,67,68,69,72,73].

Currently, four types of nanomaterials are used for wastewater treatment: dendrimeric compounds, metallic nanoparticles, zeolites and respectively carbon-based nanomaterials.

A recent development in nanomaterials means that they are especially effective in removing heavy metals (ferrite, magnetite or zinc oxide). A particular interest was paid to magnetic nanoparticles due to their properties and large specific surface area, which determines unique adsorption properties that allow easily removing the pollutants from wastewater [1,2,3,4,5,6,7,8,9,10,11,12,13,14,15,16,17,18,19,20,21,22,23,24,25,26,27,28,29,30,31,32,33,34,35,36,37,38,39,40,41,42,43,44,45,46,47,48,49,50,51,52,53,54,55,56,57,64,65,66,67,68,69,74,75,76,77].

Most research in the wastewater decontamination field used semiconductor materials to obtain various photocatalysts. There are limited studies on the use of semiconductors as adsorbents [72,75,76,77,78,79,80,81,82].

The literature describes different studies on zeolite and magnetite nanoparticles, titanium dioxide, and zinc oxides adsorbent capacity to remove pollutants from wastewater (heavy metals, compounds with nitrogen, dyes, organic compounds) [72,73,74,75,76,77,78,79,80,81,82].

The use of adsorbent materials with high efficiency in removing phosphorus can be a simple and more environmentally friendly option than the chemical method. A particularly important aspect of this is the synthesis method efficiency. So far, phosphorus removal/recovery studies have demonstrated the increased capacity of magnetic nanomaterials. However, they have the disadvantage of the high cost of synthesis. Therefore, cheap and very efficient adsorbents represent the essential condition for a possible large-scale technical implementation of this method in wastewater treatment plants. In this study, a comparative evaluation of phosphorus removal capacity and efficiency of three different adsorbents—magnetic materials (magnetite, cobalt ferrite), semiconductors (zinc oxide, titanium dioxide) and composite material (mordenite zeolite)—was conducted for the first time. Subsequently, series of experiments have been conducted to explore the effect of adsorbent dosage, and the contact time as well as the adsorption isotherm were also evaluated.

Furthermore, studies on the use of cobalt ferrite for phosphorus removal are relatively few and mainly refer to various materials that contain cobalt ferrite. To our best knowledge, this study is the first to report on the possible use of cobalt ferrite to remove phosphorus from wastewater [83,84,85].

Another novelty of this study is that, for the first time, these materials are evaluated together with two classical reagents used for phosphorus precipitation. Each of the materials selected for this study is environmentally friendly and allows the simultaneous removal of other organic or biological pollutants (zeolite, ZnO, TiO_2_) and even the possibility of recovery and reuse of phosphorus in the case of magnetic materials. The adsorbents’ phosphorus removal capacity under similar operational conditions with coagulation agents was investigated using synthetic wastewater.

The proposed materials were characterized in terms of composition and specific surface area. Subsequently, a series of experiments have been conducted to explore the effect of adsorbent type, dosage and contacting time on the adsorption efficiency.

## 2. Materials and Methods

### 2.1. Chemicals

All used reagents and solvents were analytical grade and were acquired from commercial sources (Merck, Darmstadt, Germany, WWR, Wien, Austria, Sigma-Aldrich, Darmstadt, Germany) and used without a further purification. Magnetite (nanoparticle size: 23 nm) was offered by the National Research & Development Institute for Non-ferrous and Rare Metals, Romania. Mordenite zeolite (chemical structure: (Na_2_,Ca,K_2_)_4_(Al_8_Si_40_)O_96_·28H_2_O, nanoparticle size: 42 nm and specific surface area > 400 m^2^/g) was obtained from Clariant International, Switzerland ZnO (nanoparticle size: 37 nm). Titanium dioxide nanoparticle (nanoparticle size: 54 nm) and cobalt ferrite nanoparticles (nanoparticle size: 16 nm) were offered by the Research Institute for Renewable Energies, Timisoara.

In this study, a synthetic phosphorus solution was prepared for the adsorption test. A stock phosphorus solution of 400 mg/L was prepared by dissolving the chemically pure phosphate salt (Na_2_HPO_4_∙2H_2_O) in an appropriate amount of distilled water. The pH of the solution was adjusted until at pH 7.5. Standard 0.1 M HCl and 0.1 M NaOH solutions were used for pH adjustment.

### 2.2. Experimental Procedure

The specific procedure used to investigate the influence of different types of materials was as follows: For each set of analysis, five different samples (S_1_ = 0.01 g; S_2_ = 0.03 g; S_3_ = 0.05 g; S_4_ = 0.07 g and respectively S_5_ = 0.1 g) were weighed from every type of material: coagulants (FeCl_3_ and AlCl_3_) or adsorbents (cobalt ferrite, maghemite, zinc oxide, titanium dioxide and zeolite). To each material sample (S_1_–S_5_) 10 mL of sodium phosphate solution (synthetic wastewater) was added. The resulting suspended solutions were stirred at room temperature (23 °C) for 24 h. Then, they were centrifuged, decantated and filtered (Φ185 mm filter paper), and solid residues obtained were dried in an oven at 105 °C for 24 h. Initial and final phosphate concentration after the precipitation or adsorption were analyzed using the colorimetric method with ammonium molybdate and ascorbic acid on a UV-VIS-NIR spectrometer (950 Lambda UV-Vis-NIR Perkin Elmer) at a wavelength of 880 nm [71,86]. All testing was performed with water at room temperature and a pH 7.5 [87].

The dry residues from each sample were characterized by SEM/EDAX scanning electron microscope (Quanta 400 FEG, FEI, Holland) to determinate the presence of phosphorus on the surface of materials investigated. The resulting solutions after filtration were centrifuged (450 r/min). UV–Vis analysis was conducted using a UV-VIS-NIR spectrometer (950 Lambda UV-Vis-NIR Perkin Elmer, Waltham, MA, USA) to determine the efficiency of phosphate precipitation versus adsorption using different materials [87]. In order to obtain reproducible experimental results, the adsorption experiments were carried out at least 3 times and averaged, and the obtained data and the result is accurate to 0.01%.

Statistically significant differences between the adsorbents used in this study: magnetic materials (magnetite, cobalt ferrite), semiconductors (zinc oxide, titanium dioxide) and composite (mordenite zeolite) were conducted using analysis of variance (ANOVA). The level of accepted statistical significance was *p* < 0.001 [88].

All standard phosphorus solutions with the selected concentration were prepared by diluting the stock phosphorus solution (synthetic wastewater, 400 mg/L) with distilled water.

The UV-VIS calibration curve of eight standards was plotted in the phosphorus concentration range of 0.0 mg/L and 2.0 mg/L (0.25 mg/L, 0.50 mg/L, 0.75 mg/L, 1.00 mg/L, 1.25 mg/L, 1.50 mg/L, 1.75 mg/L and 2.0 mg/L) at wavelength 880.00 nm. The correlation coefficient R^2^ = 0.9951 demonstrates the accuracy of this linear line. Function: Y = 0.5431X + 0.0209.

### 2.3. Effect of Adsorbent Dosage

To determine the effect of adsorbent dosage, we carried out a series of experiments. For each set of analysis, five different samples (S_1_ = 0.01 g; S_2_ = 0.03 g: S_3_ = 0.05 g; S_4_ = 0.07 g and respectively S_5_ = 0.1 g) from every type of adsorbent material were weighed. To each adsorbent sample (S_1_–S_5_), 15 mL phosphorus solution with concentration of 400 mg/L was added. The mixture was stirred at 200 rpm at room temperature for 24 h and then filtered (0.45 μm). The final phosphorus concentration was analyzed using the colorimetric method with ammonium molybdate and ascorbic acid on a UV-VIS-NIR spectrometer (950 Lambda UV-Vis-NIR Perkin Elmer) at a wavelength of 880 nm [86]. In order to obtain reproducible experimental results, the adsorption experiments were carried out at least 3 times.

### 2.4. Adsorption Isotherms

Freundlich and Langmuir isotherms were selected to evaluate the five selected materials’ phosphorus adsorption behavior. A batch equilibrium adsorption test was conducted as follows: in 50 mL covered Erlenmeyer flasks, 25 mL working solution and 0.05 g of adsorbent with various phosphate concentrations (5–400 mg/L) was stirred at 180 rpm for 24 h to ensure approximate equilibrium. After phosphorus adsorption, the solution was filtered through a 0.45 μm membrane filter and then analyzed for P concentration.

Langmuir and Freundlich models were used for the analysis of adsorption isotherms (Equations (1) and (2)).

(1) $$Ae_{\;}=\frac{Q_{m}-K_{L}{\;C}_{e}}{1+K_{L}C_{e}}$$

(2) $$Ae_{\;}={K_{F}K_{L}}^{1/n}$$

where A_e_ represents the adsorption removal capacity (mg/g), Ce is the phosphorus concentration in the solution at equilibrium (mg/L), Q_m_ is maximum adsorption capacity in Langmuir isotherm, K_L_ = adsorption constant in Langmuir isotherm, K_F_ = the constant of Freundlich isotherm, *n* = heterogeneity factor of Freundlich isotherm.

Phosphorus adsorption capacities and phosphorus removal efficiency, R, (%) were determined from the Equations (3) and (4) respectively:

(3) $$q=\frac{V\left(C_{0}-C_{e}\right)}{m}\;(\mathrm{mg}\;P/g)$$

(4) $$P_{r}\;(\%)=1-\frac{C_{e}}{C_{0}}\;100\%$$

where V (mL) represents the working solution volume, C_0_ and C_e_ (mg/L) are the concentrations in the working solution (initial phosphorus concentration) and filtrate (equiibrum phosphorus solution), respectively, and m (g) is the adsorbent amount [89].

Furthermore, using the Langmuir model, it was determined that the separation factor, R_L_, from the next Equation (5) is as follows:

(5) $$R_{L}=\frac{1}{1+K_{L}C_{0}}$$

(C_0_ is the initial phosphorus concentration).

### 2.5. Phosphorus Adsorption Capacity

The specific procedure was as follows: 0.1 g of each adsorbent material was weighed into a 50 mL Erlenmeyer flask, 15 mL of a set concentration of phosphorus solution was added, and the pH of the solution was adjusted to 7.5 with 0.1 mol/L hydrochloric acid or 0.1 mol/L sodium hydroxide solution. The solution was shaken in a constant temperature shaker at 180 r/min and 25 °C for 24 h. A syringe was used to take out 10 mL of the supernatant from the Erlenmeyer flask and filter it with a 0.45 µm filter membrane; the phosphorus concentration in the filtrate was determined by ammonium molybdate spectrophotometry (950 Lambda UV-Vis-NIR Perkin Elmer, Waltham, MA, USA), and the removal capacity of phosphorus and phosphorus removal efficiency of each adsorbent was calculated according to Equations (3) and (4) [83,89]. A single-factor analysis method was used and blank and two parallel experiments were carried out.

### 2.6. Effect of Reaction Time on Adsorption Capacity

For each adsorbent material the following procedure was used: 150 mL of a 400 mg/L phosphate solution was mixed with 0.1 g of adsorbent (adsorbent to solution ratio = 1:25) size in a series of 200 Erlenmeyer flasks. The pH of solution was 7.5 (adjusted with 0.1 mol/L hydrochloric acid or 0.1 mol/L sodium hydroxide solution) [83,89]. The flasks were kept at room temperature (25 °C) and 180 rpm, and samples were collected at different time intervals; phosphorus concentration was determined by ammonium molybdate spectrophotometry, and the corresponding removal capacity was calculated [83]. The calculation formula used to determine the phosphorus removal capacity (mg/g) is Equation (3).

### 2.7. Effect of Contact Time on Phosphorus Removal Efficiency 

The effect of contact time on phosphorus adsorption was observed for each adsorbent material according to the following procedure: in five different Erlenmeyer flasks (250 mL) 0.1 g adsorbent material was added; the phosphorus solution was added in 15 mL phosphorus solution with concentration of 400 mg/L and pH 7.5 (adjusted with 0.1 mol/L hydrochloric acid or 0.1 mol/L sodium hydroxide solution) [83]. The flasks were kept at room temperature (25 °C) and 180 rpm, and samples were collected at different times (0–400 min). After the water sample was left and centrifuged, the phosphorus content in the solutions was determined by ammonium molybdate spectrophotometry, and the corresponding removal capacity was calculated [83]. The calculation formula used to determine the removal efficiency (%) is Formula (4).

## 3. Material Characterization

*Nitrogen adsorption/desorption isotherms* were recorded at 77K on a NOVA 2200 apparatus. The specific surface area was calculated by Brunauer–Emmett–Teller (BET) theory from multi-point regression in the 0.08–0.3 relative pressure range [85,90,91]. The results are shown in Table 1.

### 3.1. UV-VIS Spectroscopy

The evaluation of the efficiency of each material used for phosphorus removal was done by Perkin Elmer, Lambda UV-Vis spectroscopy 950 (colorimetric method with ammonium molybdate). Phosphate reacts with ammonium molybdate in the presence of a reducing agent (ascorbic acid) to form a colored complex (blue), the intensity of which is directly proportional to the concentration of phosphate in the solution to be analyzed. The color was measured at 800 nm [86].

### 3.2. Energy-Dispersive Spectrometry (EDS)

The study of adsorbents morphology was performed through scanning electron microscopy (FEI Quanta 250 FEG) using the energy dispersive X-ray analysis detector (EDX). EDX facility was used for the semiquantitative elemental analysis.

## 4. Results and Discussions

### 4.1. Characterization of Adsorbents before and after P-Adsorbtion

#### 4.1.1. Specific Surface Area

Table 1 lists the specific surface area of each adsorbent used in this study. The specific surface area values determined experimentally correspond with the data in the literature [72,73,74,78,79,80,82,83,84,85,90,91,92,93,94,95,96,97].

#### 4.1.2. Energy Dispersive X-ray Analysis

**Figure 1:**
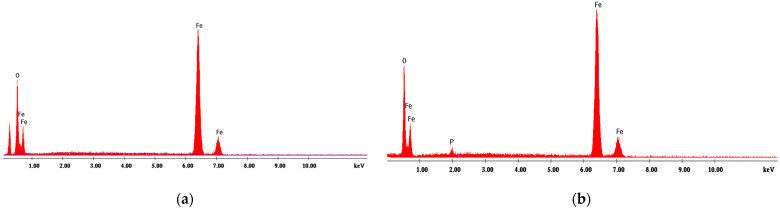
EDX analysis of magnetite (**a**) before and (**b**) after phosphorus adsorption.

**Figure 2:**
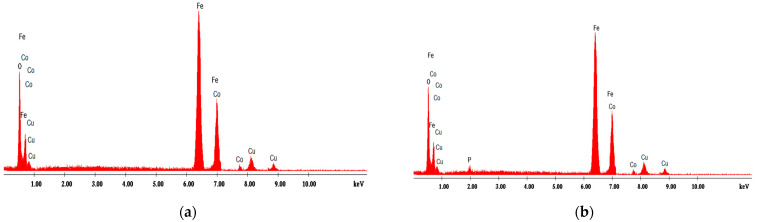
EDX analysis of cobalt ferrite (**a**) before and (**b**) after phosphorus adsorption.

**Figure 3:**
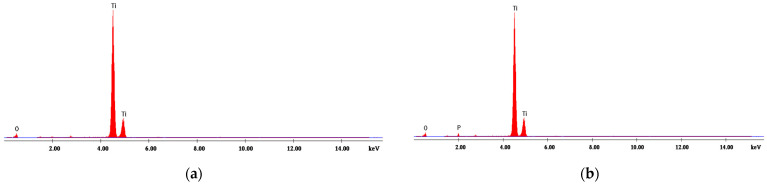
EDX analysis of titanium dioxide (**a**) before and (**b**) after phosphorus adsorption.

**Figure 4:**
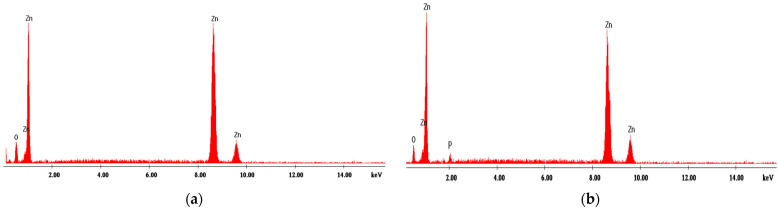
EDX analysis of zinc oxide (**a**) before and (**b**) after phosphorus adsorption.

**Figure 5:**
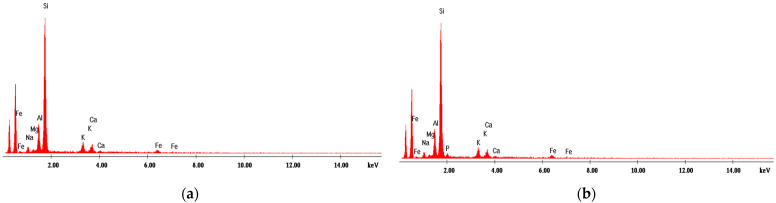
EDX analysis of zeolite (**a**) before and (**b**) after phosphorus adsorption.

Figure 1, Figure 2, Figure 3, Figure 4 and Figure 5 present EDX graphs of the adsorbents before (Figure 1a, Figure 2a, Figure 3a, Figure 4a and Figure 5a) and after the phosphorus adsorption (Figure 1b, Figure 2b, Figure 3b, Figure 4b and Figure 5b). The EDAX spectrum of adsorbents’ particles after phosphate adsorption were also analyzed, and the P element occurred in the each EDAX spectrum (Figure 1b, Figure 2b, Figure 3b, Figure 4b and Figure 5b), indicating that it was fixed on the surface of the adsorbents after phosphorus adsorption.

Comparative experimental studies have been performed on the conventional (precipitation) and alternative dephosphorization method (adsorption). The chemical precipitation of phosphorus was performed comparatively by means of two usual reactants in the tertiary stage of wastewater treatment: ferric chloride and aluminum chloride respectively. In parallel, the phosphorus removal capacity has been studied on three different types of materials: magnetic (cobalt ferrite, magnetite), semiconductors (zinc oxide, titanium dioxide) and composite (zeolite). 

Two different methods were proposed to determine the phosphorus removal performance efficiency of these materials: the colorimetric assay method and a chemical microanalysis technique based on energy dispersive X-ray spectroscopy (EDX). 

#### 4.1.3. Investigation Adsorbents Performance through UV-Vis Spectroscopy

The colorimetric assay method based on molybdenum blue phosphorus is a sensitive and highly accurate technique [86,98]. The determination of phosphorus was performed by the colorimetric method with ammonium molybdate. Phosphorus reacts with ammonium molybdate in the presence of the reducing agent (ascorbic acid solution) to form a blue complex. The blue color intensity of which is directly proportional to the content of phosphorus in final wastewater solutions (after adding coagulants or adsorbents) measured with UV-Vis spectrometer. 

#### 4.1.4. Influence of Magnetic Materials on Phosphorus Removal

**Figure 6:**
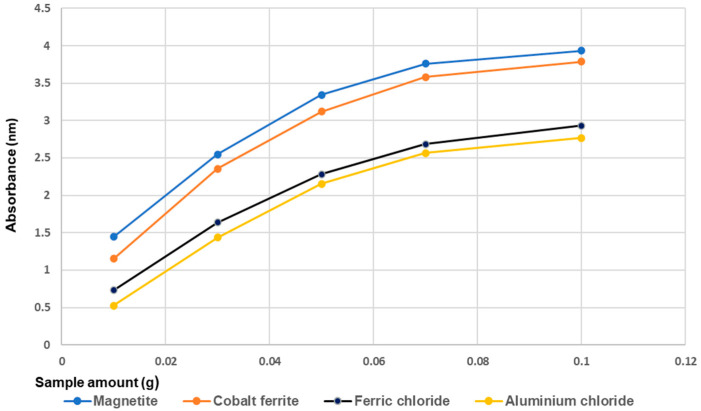
Comparison of different methods (coagulation agents vs adsorption on magnetic materials) on phosphorus removal performance in wastewater.

In the next figure (Figure 6), the influence of the conventional method (chemical precipitation with AlCl_3_ and FeCl_3_) versus adsorption on magnetic materials (cobalt ferrite and magnetite) is shown.

The results show that the highest performance of phosphorus removal was achieved for adsorption method with magnetite. The lowest absorbance value was achieved with the aluminum chloride. According to the Figure 6, the phosphorus removal performance of cobalt ferrite was slightly lower than that of magnetite. This is due to the difference between the specific surfaces of the two magnetic materials used in this study. Magnetite has a specific surface area higher than that of cobalt ferrite (Table 1) and consequently has more adsorption sites than this [90,99].

#### 4.1.5. Influence of Semiconductor Materials on Phoshorus Removal Performance

**Figure 7:**
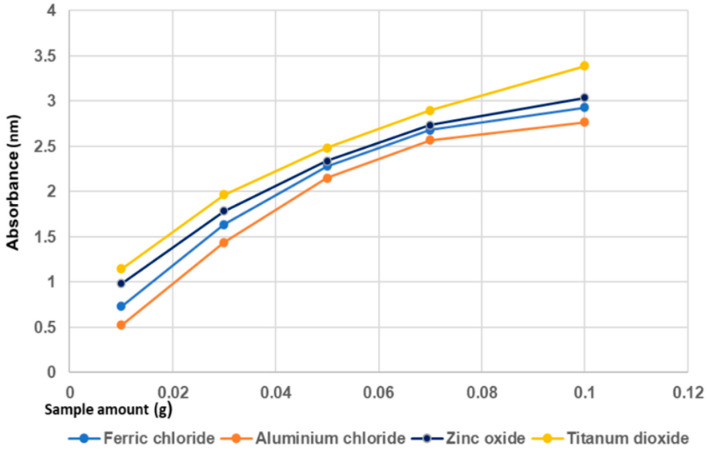
Comparison of different method (coagulation agents vs. adsorption on semiconductors materials) on phosphorus removal performance from wastewater.

In Figure 7, the comparative results of phosphorus removal performance obtained for coagulant agents and semiconductor type adsorbents (zinc oxide/titanium dioxide) are presented.

The results show that the highest performance of phosphorus removal was achieved for the adsorption method with titanium dioxide. And the lowest yield was achieved with the aluminum chloride. According to Figure 7, the phosphorus removal performance of zinc oxide was slightly lower than of titanium dioxide. This is due to the difference between the specific surfaces of the two semiconductor materials used in this study. The specific surface area of titanium dioxide is about three times larger than that of zinc oxide (Table 1). This may explain the difference of phosphorus removal performance between these semiconductors [75,86,97].

#### 4.1.6. Influence of Composite Material on Phosphorus Removal Performance

**Figure 8:**
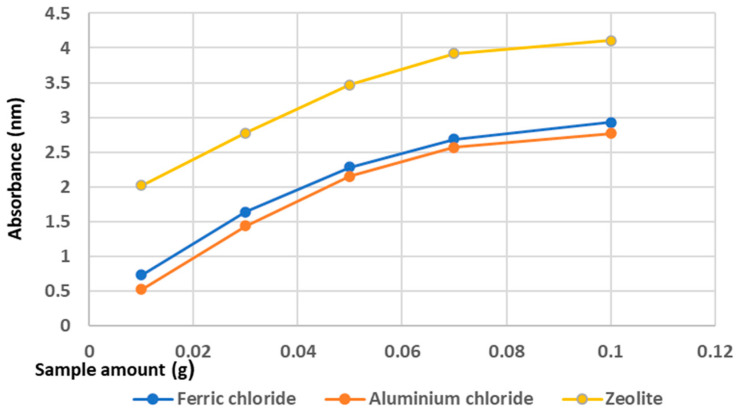
Comparison of different method (coagulation agents vs adsorption on composite materials) on phosphorus removal performance from wastewater.

The difference of phosphorus removal performance between the coagulation agents and adsorption on mordenite zeolite is depicted in Figure 8.

It can be seen from Figure 8 that phosphorus adsorption on zeolite was more efficient than both conventional coagulation agents used in this study. It is well known that zeolite is one of the most effective adsorbents. In fact, it is used successfully in the process of removing various heavy metals or nitrogen compounds from wastewater. Moreover, the crystalline, highly porous structure of zeolite represent key factors in adsorption process efficiency [81,95,96].

#### 4.1.7. Effect of Different Type of Adsorbents on Phosphorus Removal Performance

**Figure 9:**
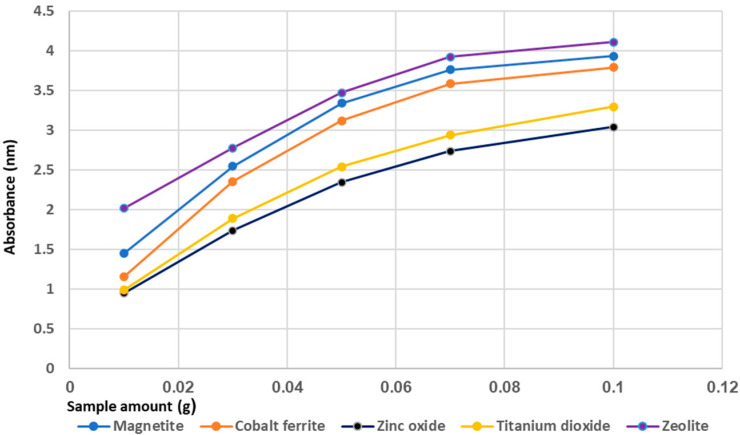
Comparison of different adsorbents used for phosphorus removal.

To investigate how the performance of the phosphorus adsorption process depended on the absorbers’ type, a series of adsorption experiments were conducted on three distinct categories of materials: magnetic, semiconductors and composite (Figure 9).

The adsorption curves shown in Figure 9 indicate that the yield of the adsorption is directly proportional to the specific surface area of the adsorbent material. It is known that a material with a large specific surface area can act as an effective adsorbent. And according to the adsorption curves depicted in Figure 9, this fact is confirmed. Thus, the best efficiency was obtained for zeolites, followed by magnetite and cobalt ferrite. And the lowest performance was for zinc oxide. The phosphorus adsorption performance for TiO_2_ was slightly higher than for zinc oxide.

#### 4.1.8. Effect of Adsorbent Dosage

**Figure 10:**
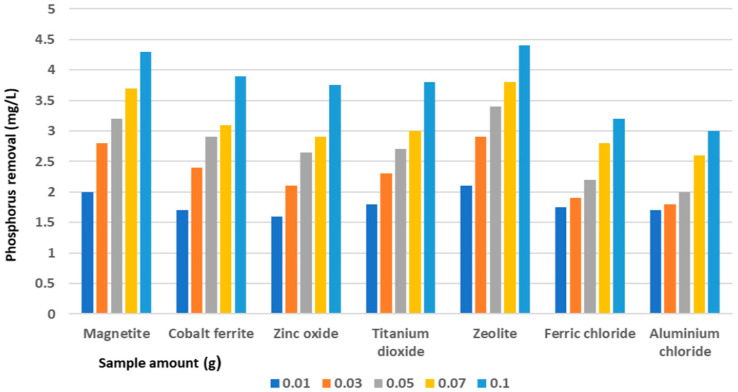
The effect of adsorbent dosage on phosphorus removal.

The influence of adsorbent dosage is shown in Figure 10. The adsorbent mass was varied from 0.01 g to 0.1 g.

It was found that increasing the adsorbent dosage lead increased the P-adsorption removal yield. The maximum yield was obtained for mordenite zeolite, followed by magnetic materials semiconductors and coagulation agents.

These results suggest that the optimal dosage of adsorbents can efficiently remove phosphorus from wastewater. At the same time, it interesting to note that magnetic materials could favorably influence the effectiveness of phosphorus recovery and reuse as a fertilizer in agriculture. A high dosage addition of the other adsorbents used, such as zinc oxide, titanium dioxide or zeolite, could be beneficial considering their antibacterial properties.

### 4.2. Adsorption Isotherms

The Langmuir and Freundlich models were employed to obtain information on the characteristics of the adsorption process as well as on the optimum use of each adsorbent used in this study. Table 2 summarizes the results.

The results indicate that both Langmuir and Freundlich models fitted the experimental data well. Nevertheless, the higher value of the correlation coefficient R^2^ is shown by the Langmuir model, which suggests that this model is more appropriate to describe the adsorption on the five materials investigated in the study (magnetite, cobalt ferrite, titanium dioxide, zinc oxide and mordenite zeolite). Simultaneously, the constant n in Freundlich model has super unit values for all five adsorbents used, indicating that the phosphorus adsorption is nonlinear. Moreover, the separation factor values, R_L_, for all adsorbents used in these studies falls within the range 0.00101 < R_L_ < 0.00322 indicated the favorability of the adsorption process [85,100].

**Figure 11:**
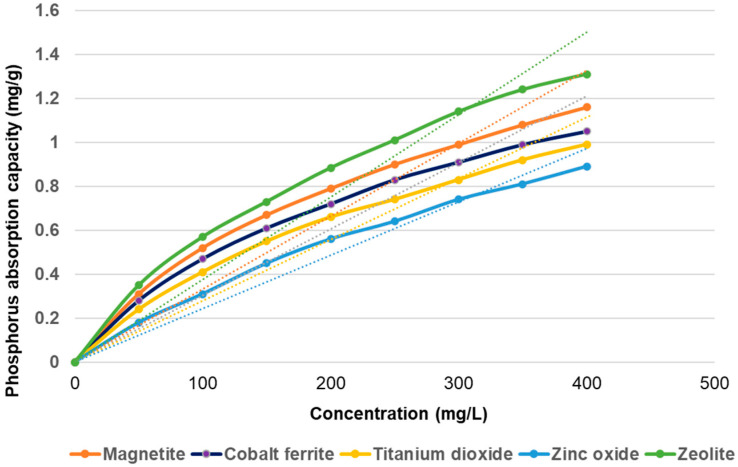
The adsorbents Langmuir and Freundlich isotherms.

The adsorption isotherms of the three types of materials investigated in this study are presented in Figure 11.

The results suggest that the phosphorus adsorption capacity increases directly in proportion to the adsorbent concentration. A maximum adsorption capacity of 1.31 mg/g of the composite material (zeolite) used was obtained at the initial phosphorus concentration of 400 mg/L. The adsorption capacity of magnetite at the same experimental condition is 1.16 mg/g, and the lowest value (0.89 mg/g) was obtained for zinc oxide.

#### 4.2.1. ANOVA Test

Table 3 presents the results of the ANOVA single factor test.

The results (F = 8.015178 and *p* = 1.57 × 10^−4^) showed a value for F higher than 4, and the *p*-value is lower than 0.05, which suggests that the differences in in phosphorus removal (mg P/g) differed significantly across the adsorbents used [100].

#### 4.2.2. Phosphorus Adsorption Capacity

**Figure 12:**
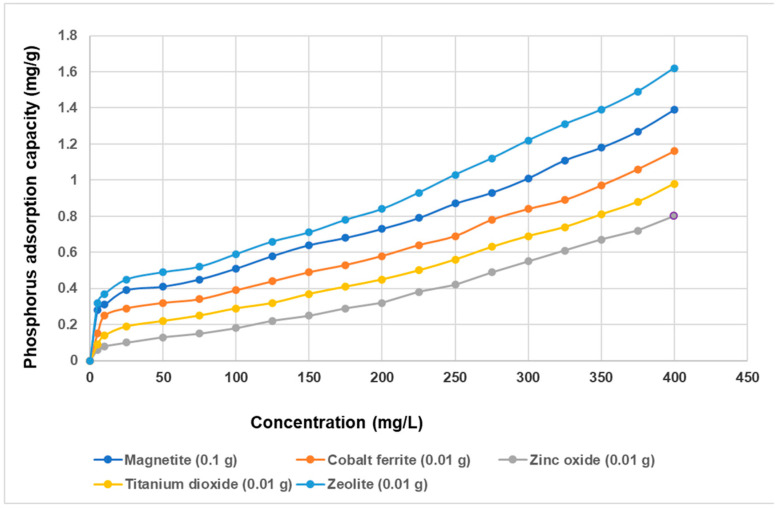
Relationship between initial concentration and adsorbents’ phosphorus adsorption capacity.

The relationship between initial concentration and adsorbents’ phosphorus adsorption capacity is depicted in the Figure 12.

As can be seen from Figure 12, with the increase in the initial phosphorus concentration, the overall adsorption capacity of adsorbents showed an upward trend. Compared to the previous figure (Figure 11), a slight increase in the adsorption capacity manifested by all adsorbents investigated was found. This can be explained by the fact that it worked with a double amount of adsorbent (0.1 g) and thus has more adsorption sites [101].

Thus, at the initial phosphorus concentration of 400 mg/L., it was found that the maximum adsorption capacity of zeolite is 1.58 mg/g, followed by magnetite with a maximum of 1.39 mg/g. And, in this case, the semiconductor materials confirmed that they have a lower phosphorus adsorption capacity compared to the zeolite and magnetic materials used. However, from the shared analysis of the two figures (Figure 11 and Figure 12), a small variation of the maximum of the adsorption capacity in the case of semiconductor materials was found even under conditions in which the initial amount of adsorbent was higher. These results indicate that the adsorption is dependent on the chemical composition and morpho-structural properties of the adsorbent (specific surface, porosity) [85,90].

### 4.3. Effect of Time of P-Removal Capacity of Adsorbants

**Figure 13:**
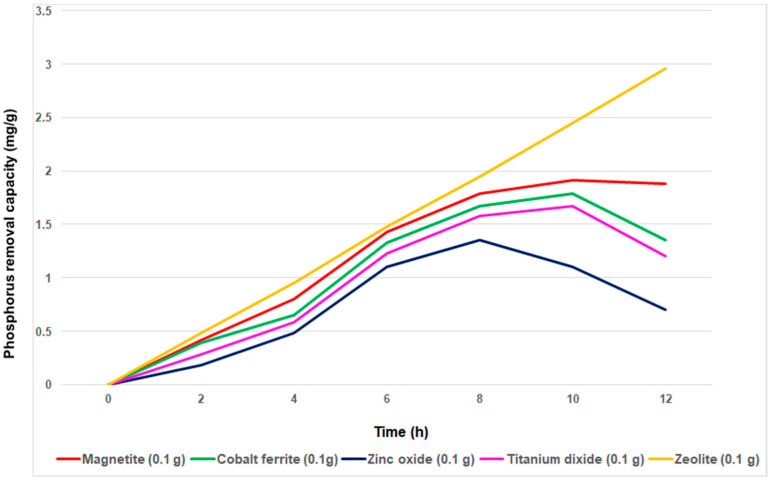
The effect of contacting time on the removal capacity of absorbents.

Figure 6, Figure 7, Figure 8, Figure 9 and Figure 10 show that for all adsorbent materials, the best performance was obtained at the highest amount used (0.1 g). In order to determine the effect of contacting time on P-adsorption, further experiments were carried out using 0.1 g of each adsorbent, at room temperature and pH 7.5, with the contacting time varying in the range of 2–12 h (Figure 13).

Figure 13 shows the effect of contacting time on the phosphorus removal capacity of magnetite, cobalt ferrite, titanium dioxide, zinc oxide and mordenite zeolite. In the early stage, the removal capacity of each absorbents increases. And then, after 8 h, the removal capacity of three of the adsorbents evaluated (magnetite, cobalt ferrite and titanium dioxide) reach the set stage, and after 10 h, a slight decrease occurs. However, zinc oxide removal capacity was slightly lower than that of the other adsorbents in the whole range of time investigated. It can also be observed that only the removal capacity of zeolite was extremely high and remained constant in the whole range of the time investigated. Hence, prolonged time may promote the access of ions to active sites on the surface of adsorbent. And it is obvious that the efficiency of phosphorus removal will decrease in proportion to the adsorbents’ specific surface. Under the above-mentioned conditions, the maximum removal capacity was obtained for zeolite followed by magnetite, cobalt ferrite, titanium dioxide and zinc oxide.

### 4.4. Effect of Contact Time on Phosphorus Removal Efficiency

**Figure 14:**
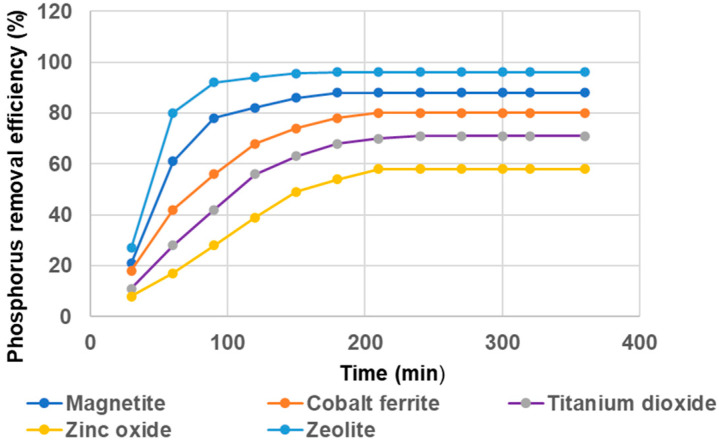
Effect of contact time on phosphorus adsorption efficienc.

The variation in phosphorus removal efficiency with contact time at 400 g/L initial phosphorus concentration is presented in Figure 14.

It may be observed from the figure above (Figure 14) that for zeolite and magnetite, the rapid phosphorus adsorption took place within 90 min; then adsorption becomes slow and almost attains equilibrium after 150 min. Significant change in the extent of adsorption was not observed (>1%) for an even further increase in contact time of up to 360 min. Instead, in the case of the other materials used, (cobalt ferrite, titanium dioxide and zinc oxide) it can be it can be seen that there is a rapid adsorption that takes place up to 150 min, after which the process becomes very slow close to equilibrium and only >4% changes in adsorption efficiency occur. Therefore, the rest of the experiments were conducted for 3600 min. A total of 96% adsorption for zeolite was obtained, and 88% for magnetite. The phosphorus percentage removal decreases for semiconductors: 71% for titanium dioxide and only 58% for zinc oxide. This indicates that the rate of phosphorus removal increases rapidly in proportion to the specific surface area of the material, and as time proceeds, the number of active sites were decreased and then ad-sorption becomes increasingly slowed down.

## 5. Conclusions

In the present study, the possibility of removing phosphorus from wastewater using nanomaterials or composite materials was investigated: magnetite, cobalt ferrite, zinc oxide, titanium dioxide and mordenite zeolite under identical experimental conditions. The efficacy of some adsorbents (selected for this study) and conventional coagulation agents used in the tertiary wastewater treatment were compared. The effects of different reaction parameters: the initial phosphorus concentrations, reaction time, adsorbents dosage on phosphorus adsorption capacity and efficiency were investigated through different analytical techniques: UV-Vis spectroscopy, BET and EDS analyses. Present results suggest that the efficiency of the adsorbent of phosphorus removal was superior compared to ferric chloride and aluminum chloride. Under the experimental conditions (temperature (23 °C) with pH 7.5), phosphorus adsorption was strongly dependent on the surface area of adsorbent, adsorbent dosage and contact time. Mordenite zeolite and magnetic materials exhibit high phosphorus adsorption capacity and adsorption efficiency compared with the semiconductor materials used in this study. The phosphorus adsorption efficiency decreases in the following order: composite material (mordenite zeolite 96%), magnetic materials (magnetite 88%, cobalt ferrite 80%) and semiconductor materials (titanium dioxide 71% and 51% zinc oxide). At room temperature (25 °C), both the Langmuir and Freundlich equation can reasonably describe the adsorption, and the maximum adsorption capacity fitted by the Langmuir equation was 1.31 mg/g for zeolite, which decreased at 1.16 mg/g for magnetite, 1.05 mg/g for cobalt ferrite, 0.99 mg/g for titanium dioxide and 0.89 mg/g for zinc oxide. The proposed adsorbents present the possibility of recovering phosphorus and reusing it as a fertilizer (adsorbents of a magnetic nature) or other organic or biological pollutants removal (TiO_2_, ZnO and zeolites). Adsorption performance analysis can provide valuable information, representing the practical value of these materials as an effective, convenient alternative within the circular economy model for water pollution mitigation and management.

## Figures and Tables

**Table 1:** Material specific surface area determinate by Brunauer–Emmett–Teller theory (BET).

Absorbent Type	Cobalt Ferrite	Magnetite	Zinc Oxide	Titanium Dioxide	Mordernite Zeolite
Specific Surface Area (m^2^/g)	110	130	29.7	48.25	413.2

**Table 2:** The adsorbents Langmuir and Freundlich isotherm parameters.

Adsorbent Material	Langmuir Model			Freundlich Model		
	Q_m_	K_L_	R^2^	Kn	*n*	R^2^
Magnetite	3.966	0.00128	0.990	0.0164	1.381	0.988
Cobalt ferrite	3.387	0.00124	0.981	0.0141	1.388	0.977
Titanium dioxide	3.317	0.00113	0.978	0.0138	1.327	0.974
Zinc oxide	3.275	0.00101	0.975	0.0092	1.277	0.973
Zeolite	3.898	0.00322	0.998	0.0172	1.395	0.996

**Table 3:** Statistical parameters obtained from the ANOVA test.

Source	Sum of Square (SS)	Degree of Freedom (df)	Mean Squares (MS)	F	*p*-Value	F Crit
Between Groups	3.165918	4	0.791479	8.015178	1.57 × 10^−4^	2.479015
Within Groups	8.393544	85	0.098748	-	-	-
Total	11.55946	89	-	-	-	-

## Data Availability

All data are contained within the article.

## References

[1] Breisha G.Z. (2010). Bio-removal of nitrogen from wastewaters—A review. Nat. Sci..

[2] Butusov M., Jernelöv A. (2013). Phosphorus: An Element That Could Have Been Called Lucifer.

[3] Cornel P., Schaum C. (2009). Phosphorus recovery from wastewater: Needs, technologies and costs. Water Sci. Technol..

[4] Czemiel-Berndtsson J. (2004). Urban Wastewater Systems: From Disposal to Reuse—An Analysis of the Performance of Different Systems with Focus on Water and Nutrients Flows. Ph.D. Thesis.

[5] Damian C., Barbul A. (2000). Tehnologii noi Pentru Încadrarea în Normativul NTPA 001, Nitrificare, Denitrificare, Defosforizare în Treapta Biologică, Seminar Ştiinţific–Tehnologii Pentru Reţinerea Azotului şi Fosforului din Apele Uzate şi Necesitatea Dezinfectării Apelor Epurate.

[6] Department of the Army (2001). Biological Nutrient Removal, Public Works Technical Bulletin 420-49-39.

[7] Faby J.A., Brissaud F., Bontoux J. (1999). Wastewater reuse in France: Water quality standards and wastewater treatment technologies. Water Sci. Technol..

[8] Gubin S.P., Koksharov Yu A., Khomutov G.B., Yurko G.Y. (2005). Magnetic nanoparticles: Preparation, structure and properties. Russ. Chem. Rev..

[9] Heckenmüller M., Narita D., Klepper G. (2014). Global Availability of Phosphorus and Its Implications for Global Food Supply: An Economic Overview.

[10] Van Kauwenbergh S. (2010). World Phosphate Rock Reserves and Resources, IFDC Technical Bulletin 75.

[11] Marchal V., Dellink R., van Vuuren D., Clapp C., Château J., Lanzi E., Magné B., van Vliet J. (2011). OECD Environmental Outlook to 2050, Climate Change Chapter, Pre-Release Version.

[12] Margeta K., Zabukovec Logar N., Šiljeg M., Farkaš A., Elshorbagy W., Chowdhury R.K. (2013). Natural Zeolites in Water Treatment–How Effective Is Their Use, Water Treatment.

[13] National Primary Drinking Water Regulations EPA United States Environmental Protection Agency. http://water.epa.gov/drink/contaminants/index.cfm.

[14] Ngo H.H., Guo W., Surampalli R.Y., Zhang T.C. (2016). Green Technologies for Sustainable Water Management.

[15] Robescu D., Verestoy A., Lányi S., Robescu L.D. (2002). Fiabilitatea Proceselor, Instalaţiilor şi Echipamentelor de Tratare şi Epurarea Apei.

[16] Sedlak R.I. (1991). Phosphorus and Nitrogen Removal from M unicipal Wastewater, Principles and Practice.

[17] Stark K., Plaza E., Hultman B. (2006). Phosphorus release from ash, dried sludge and sludge residue from supercritical water oxidation by acid or base. Chemosphere.

[18] Statutory Instruments S.I. (2007). No. 106, European Communities (Drinking Water) Regulations. http://www.attorneygeneral.ie/esi/2007/B25111.pdf.

[19] Syers K., Bekunda M., Cordell D., Corman J., Johnston J., Rosemarin A., Salcedo I., Lougheed T. (2011). UNEP Year Book, Phosphorus and Food Production.

[20] Tan C.M., Kong J.C. (2010). FTIR spectroscopy as a tool for nano-material characterization Infrared. Phys. Technol..

[21] Tchobanoglous G., Burton F.L., Stensel H.D. (2003). Wastewater Engineering—Treatment and Reuse.

[22] Tiwari D.K., Behari J. (2008). Prasenjit Sen, Application of nanoparticles in waste water treatment. World Appl. Sci. J..

[23] UN (2010). The Millennium Development Goals Report.

[24] UN (2013). World Population Prospects: The 2012 Revision.

[25] Akpor O.B., Muchie M. (2010). Bioremediation of polluted wastewater influent: Phosphorus and nitrogen removal. Rev. Sci. Res. Essays.

[26] Banerjee S., Gautam P.K., Jaiswal A., Chattopadhyaya M.C. (2017). Recent Trends and Advancement in Nanotechnology for Water and Wastewater Treatment: Nanotechnological Approach for Water Purification. Materials Science and Engineering: Concepts, Methodologies, Tools, and Applications.

[27] Balica S.F., Popescu I., Beevers L., Wright N.G. (2013). Parametric and physically based modelling techniquesfor flood risk and vulnerability assessment: A comparison. J. Environ. Model. Softw..

[28] Begum R., Farooqi Z.H., Naseem K., Madeeha F.A., Batool J.X., Irfan A. (2018). Applications of UV/Vis spectroscopy in characterization and catalytic activity of noble metal nanoparticles fabricated in responsive polymer microgels: A Review. Crit. Rev. Anal. Chem..

[29] Cho R. (2013). Phosphorus: Essential to Life-Are We Running Out? State of the Planet Columbia University. http://blogs.ei.columbia.edu/2013/04/01/phosphorus-essential-to-life-are-we-running-out/.

[30] Clesceri L.S., Eaton A.D., Greenberg A.E. (1996). Standard Methods for the Examination of Water and Wastewater. 19th Edition Supplement, American Public Health Association, American Water Works Assocation.

[31] (2008). Cohre, WaterAid, SDC and UN-HABITAT, Sanitation: A Human Rights Imperative.

[32] (2013). Comisia Europeană Bruxelles, 8.7.2013 Com. 517 Final Comunicare A Comisiei către Parlamentul European, Consiliu, Comitetul Economic si Social European Și ComitetulRegiunilor Comunicare Consultativă Privind Utilizarea Durabilă a Fosforului.

[33] Cordell D., Drangert J.O., White S. (2009). The story of phosphorus: Global food security and food for thought. Glob. Environ. Chang..

[34] Damian D., Grozescu I., Segneanu A. (2015). Use of nanoparticles in water management. Conferinta Internationala Multidisciplinara “Profesorul Dorin Pavel Fondatorul Hidroenergeticii Romanesti”.

[35] de Graaff M.S., Temmink H., Zeeman G., Buisman C.J.N. Energy and phosphorus recovery from black water. Proceedings of the IWA 12th Wold Congress on Anaerobic Digestion.

[36] Dinh N.Q., Balica S., Popescu I., Jonoski A. (2012). Climate change impact on flood hazard, vulnerability and risk of the Long Xuyen Quadrangle in the Mekong Delta. J. River Basin Manag..

[37] Driver J., Lijmbach D., Steen I. (1999). Why recover phosphorus for recycling, and how?. Environ. Technol..

[38] Gligor E., Blaga A.C. (2009). Techniques for removing nitrogen and phosphorus through chemical addition. An. Univ. Din OradeaFascicula De Energetică.

[39] Kang S.J., Olmstead K., Takacs K., Collins J. (2008). Municipal Nutrient Removal Technologies Reference Document Vol. 1.

[40] Hermann H.H., Hoffmann E., Odegaard H. Chemical Water and Wastewater Treatment. Proceedings of the 8th Gothenburg Symposium.

[41] Holba M., Škorvan O., Maršálková E., Maršálek B. (2012). Phosphorus Removal from Wastewater via Environmentally Friendly Technologies, Nanocon, 23-25.10.

[42] Imran A. (2012). New generation absorbents for water treatment. Chem. Rev..

[43] Ionescu G.C. (2010). Sisteme de Epurare a Apelor Uzate.

[44] Klapetek P., Valtr M., Nečas D., Salyk O., Dzi P. (2011). Atomic force microscopy analysis of nanoparticles in non-ideal conditions. Nanoscale Res. Lett..

[45] (1998). Directiva 98/83/CE a Consiliului din 3 Noiembrie 1998 Privind Calitatea Apei Destinate Consumului Uman. Jurnalul Oficial al Comunităţilor Europene nr.

[46] Luo H.B., Li F., Li H., Zeng Y.M., Zhang K., Huang B. (2012). Adsorption control performance of phosphorus removal from agricultural non-point source pollution by nano-aperture lanthanum-modified active alumina. Adv. J. Food Sci. Technol..

[47] Magnaye F.A., Gaspillo P.D., Auresenia J.L. (2009). Biological nitrogen and COD removal of nutrient-rich wastewater using aerobic and anaerobic reactors. J. Water Resour. Prot..

[48] Mohamed E.A. (2020). El-sayed. Nanoadsorbents for water and wastewater remediation. Sci. Total Environ..

[49] Muste M., Quinn P.F., Hewett C.J.M., Popescu I., Basu N.B., Kumar P., Franz K., Merwade V., Arnold W., Potter K. (2010). Initiation of the Upper Mississippi River Basin Observatory. Proc. ASCE Innov. Watershed Manag. Land Use Clim. Chang..

[50] Machado A.V., Nogueira R. (2012). Phosphorus Removal from Eutrophic Waters with an Aluminium Hybrid Nanocomposite. Water Air Soil Pollut..

[51] Parsons S., Smith J.A. (2008). Phosphorus removal and recovery from municipal wastewaters. Elements.

[52] Pesetskii S.S., Bogdanovich S.P., Myshkin N.K. (2013). Polymer nanocomposites with thermoplastic matrices-Processing and Tribology. J. Macromol. Sci. Part B.

[53] Petrea N., Iordache P.Z., Lungu R.M., Petre R., Pretorian A. (2011). New methods and new types of functionalised nanocomposites intended for the ecological depollution of waters. Nanocomposites.

[54] Schmidt I., Sliekers O., Schmid M., Bock E., Fuerst J., Kuenen J.G., Jetten M.S.M., Strous M. (2003). New concepts of microbial treatment processes for the nitrogen removal in wastewater. FEMS Microbiol. Rev..

[55] UN (2012). General Assembly Resolution 66/288: The Future We Want.

[56] UN (2012). World Urbanization Prospects—The 2011 Revision—Highlights.

[57] UN (2013). Water Analytical Brief on Water Security and the Global Water Agenda.

[58] (2007). Fact Sheets on Biological Nutrient Removal Processes and and Costs.

[59] van Haandel A., van der Lubbe J. (2007). Handbook Biological Waste Water Treatment Design and Optimisation of Activated Sludge Systems.

[60] Van Kauwenbergh S.J. (2014). U.S. Department of Agriculture, Agricultural Outlook Forum 20–21 February.

[61] Van P.D.T., Popescu I., van Griensven A., Solomatine D., Trung N.H., Green A. (2012). A study of the climate change impacts on fluvial flood propagation in the Vietnamese Mekong Delta. Hydrol. Earth Syst. Sci..

[62] Vesilind P.A. (1998). Wastewater Treatment Plant Design. Water Environment Federation.

[63] Vimonses V. (2011). Development of Multifunctional Nanomaterials and Adsorbtion-Photocatalysis Hybrid System for Wastewater Reclamation. Ph.D. Thesis.

[64] WHO (World Health Organization) (2008). Health through Safe Drinking Water and Basic Sanitation. http://www.who.int/water_sanitation_health/mdg1/en/index.html.

[65] United Nations Educational, Scientific and Cultural Organization (2012). United Nation UNWater UN World Water Development Report: 2012.

[66] Abosede S., Shikuku V. (2020). Use of Low Cost Materials to Remove Chemicals of Emerging Concern from Wastewater Effluents, Effects of Emerging Chemical Contaminants on Water Resources and Environmental Health.

[67] Wu Quanguo W., Changzhong Jiang H. (2008). Magnetic iron oxide nanoparticles: Synthesis and surface functionalization strategies. Nanoscale Res. Lett..

[68] Xu P., Zeng G.M., Huang D.L., Feng C.L., Hu S., Zhao M.H., Lai C., Wei Z., Huang C., Xie G.X. (2012). Use of iron oxide nanomaterials in wastewater treatment. Sci. Total Environ..

[69] Yang K., Li Z., Zhang H., Qian J., Chen G. (2010). Municipal wastewater phosphorus removal by coagulation. Environ. Technol..

[70] Baldi M., Martinotti A., Sorlini S., Katsoyiannis I.A., Abbà A., Carnevale Miino M., Collivignarelli M.C. (2021). Extraction and purification of phosphorus from the ashes of Incinerated biological sewage sludge. Water.

[71] Kleemann R., Chenoweth J., Clift R., Morse S., Pearce P., Saroj D. (2017). Comparison of phosphorus recovery from incinerated sewage sludge ash (ISSA) and pyrolysed sewage sludge char (PSSC). Waste Manag..

[72] Schröder J.J., Cordell D., Smit A.L., Rosemarin R. (2010). Sustainable Use of Phosphorous.

[73] Zhigalina O.M., Mishina E.D., Sherstyuk N.E., Vorotilov K.A., Vasiljev V.A., Sigov A.S., Lebedev O.I., Grigoriev Y.V., De Santo M.P., Barberi R. (2006). Crystallization of PZT in porous alumina membrane channels. Ferroelectrics.

[74] Lowell S., Shields J.E., Thomas M.A., Thommes M. (2004). Characterization of Porous Solids and Powders: Surface Area, Pore Size and Density.

[75] Sadovnikov S.I., Gusev A.I. (2018). Effect of particle size and specific surface area on the determination of the density of nanocrystalline silver sulfide Ag_2_S powders. Phys. Solid State.

[76] Peshev O. (1974). Adsorption on Semiconductors in Relation to Their Degree of Dispersity. Jpn. J. Appl. Phys..

[77] Kiser M.A., Westerhoff P., Benn T., Wang Y., Pérez-Rivera J., Hristovski K. (2009). Titanium Nanomaterial Removal and Release from Wastewater Treatment Plants. Environ. Sci. Technol..

[78] Shtyka O., Shatsila V., Ciesielski R., Kedziora A., Maniukiewicz W., Dubkov S., Gromov D., Tarasov A., Rogowski J., Stadnichenko A. (2021). Adsorption and photocatalytic reduction of carbon dioxide on TiO_2_. Catalysts.

[79] Vlasova N.N., Markitan O.V. (2020). Adsorption of inorganic phosphates on a titanium dioxide surface. Colloid J..

[80] Choi S. (2016). Phosphorus Removal Using Titanium Dioxide Nanoparticles in Wastewater Treatment. Master’s Thesis.

[81] Erdem E., Karapinar N., Donat R. (2004). The removal of heavy metal cations by natural zeolites. J. Colloid Interface Sci..

[82] Compagnini G., Syzrantsev V., Paukshtis E., Larina T., Chesalov Y., Bardakhanov S., Nomoev A. (2018). Features of surface structures of alumina and titanium dioxide nanoparticles produced using different synthes. J. Nanomater..

[83] Zhao J., Gao J., Liu J. (2020). Preparation of a new iron-carbon-loaded constructed wetland substrate and enhanced phosphorus removal performance. Materials.

[84] Karthikeyan P., Vigneshwaran S., Preethi J., Meenakshi S. (2020). Preparation of novel cobalt ferrite coated-porous carbon composite by simple chemical co-precipitation method and their mechanistic performance. Diam. Relat. Mater..

[85] Dey A., Singh R., Purkait M. (2014). Cobalt ferrite nanoparticles aggregated schwertmannite: A novel adsorbent for the efficient removal of arsenic. J. Water Process Eng..

[86] Elmer P. (2015). Application note-UV/Visible spectroscopy-Water analysis using Lambda: Total phosphorus (T-P). Ascorbic Acid Method.

[87] Segneanu A.E. (2020). Advanced Materials—As an Alternative for Phosphate Pollution Removal from Wastewater. Master’s Thesis.

[88] Yin Q., Wang R., Zhao Z. (2018). Application of Mg-Al-modified biochar for simultaneous removal of ammonium, nitrate, and phosphate from eutrophic water. J. Clean. Prod..

[89] Luo Z., Zhu S., Liu Z., Liu J., Huo M., Yang W. (2015). Study of phosphate removal from aqueous solution by zinc oxide. J. Water Health.

[90] Shen Y.F., Tang J., Nie Z.H., Wang Y.D., Ren Y., Zuo L. (2009). Preparation and application of magnetic Fe_3_O_4_ nanoparticles for wastewater purification. Sep. Purif. Technol..

[91] Zhang L., Dan H., Bukasa O.T., Song L., Liu Y., Wang L., Li J. (2020). Low-cost efficient magnetic adsorbent for phosphorus removal from water. ACS Omega.

[92] Panasiuk O. (2010). Phosphorus Removal and Recovery from Wastewater Using Magnetite. Master’s Thesis.

[93] Amin M.T., Alazba A.A., Manzoor U. (2014). A Review of removal of pollutants from water/wastewater using different types of nanomaterials. Adv. Mater. Sci. Eng..

[94] Borysiewicz M.A. (2019). ZnO as a functional material, A Review. Crystals.

[95] Moshoeshoe M., Tabbiruka N.S.M., Obuseng V. (2017). A review of the chemistry, structure, properties and applications of zeolites. Am. J. Mater. Sci..

[96] Korkuna O., Leboda R., Skubiszewska-Zieba J., Vrublevs’ka T., Gun’ko V.M., Ryczkowski J. (2006). Structural and physicochemical properties of natural zeolites: Clinoptilolite and mordenite. Microporous Mesoporous Mater..

[97] (2017). Clariant-Brochure Zeolite Materials.

[98] Kharat S., Pagar S. (2009). Determination of phosphate in water samples of Nashik District (Maharashtra State, India) rivers by UV-Visible spectroscopy. J. Chem..

[99] Wu F., Yu Q., Gauvin F., Brouwers H., Liu C. (2021). Phosphorus removal from aqueous solutions by adsorptive concrete aggregates. J. Clean. Prod..

[100] Mekonnen D.T., Alemayehu E., Lennartz B. (2020). Removal of Phosphate Ions from Aqueous Solutions by Adsorption onto Leftover Coal. Water.

[101] Sun J., Gao A., Wang X., Xu X., Song J. (2020). Removal of Phosphorus from Wastewater by Different Morphological Alumina. Molecules.

